# Living on the Edge: A Cocreated Teaching-Learning Journey

**DOI:** 10.1177/08943184251374532

**Published:** 2025-10-21

**Authors:** Karen L. Ursel

**Affiliations:** 1 Teaching Professor/Clinical Strategist, Faculty of Nursing, University of New Brunswick, Moncton, Moncton, NB, Canada

**Keywords:** Giorgi’s descriptive phenomenological method, Parse’s humanbecoming paradigm, living on the edge, teaching-learning

## Abstract

Parse’s humanbecoming teaching-learning model provided the framework for guiding the analysis-synthesis in this study using Giorgi’s descriptive phenomenological method to reach the general structural description of the lived experience of *living on the edge* for nursing students. The central findings emerged as *acquiescing to ambiguity* in moving moment-to-moment coming-to-know something of value, *dignifying the journey* by vigilantly exploring unknowns of the everchanging, *risking venturing forth* with quieting-disquieting regard amid diverse alliances in anticipation of cherished endeavors, *persevering with novel experiences* amid potentiating-restricting opportunities in realizing cherished endeavors, *venerating words, wisdom, and images* of others in considering-composing cherished choosings, and *discovering anew cherished priorities* as possibilities emerge.

Creating a teaching-learning environment where clients, families, students, and staff feel valued, honored, and respected has been challenging in today’s healthcare climate. The literature on shifting teaching-learning approaches is ubiquitous, but learning experiences described from the student’s perspective are limited. Benner and colleagues (2010) challenged nurse educators to consider significant changes in their teaching approaches. In healthcare today, there are no absolutes; there is an explosion of information with discoveries and advances. Learning to practice or live the art and science of nursing requires more of the teaching-learning relationship: exploring, understanding, and appreciating these learning experiences. The focus of this study was to uncover the meaning of the lived experience of living on the edge for nursing students in a teaching-learning context, a topic of significant importance in nursing education. Giorgi’s (2009) descriptive phenomenological research method was used, and findings were viewed through the lens of Parse’s (2021a) humanbecoming paradigm and, more specifically, the humanbecoming teaching-learning model (Parse, 2004, 2021a). The humanbecoming paradigm provided the philosophical stance that informed the method and provided the discipline-specific context for the process. This view recognizes that individuals are always learning and changing within their day-to-day experiences, and this knowing is incorporated into an existing framework of experiences, values, and beliefs.

## Related Literature

The extant literature on teacher-learner pedagogies in nursing education shows a growing awareness of the significance of teacher-learner relationships in the learning journey (Tanner, 2007). Nursing education has continued to be defined in terms of learning objectives, which focus on what the teacher must provide in course content (teacher-focused performance). Today’s most common pedagogy focuses on outcomes, competency, or ability-based nursing curricula, valued by professional organizations as concrete or objective evidence that students are safe to practice according to professional standards (learner-focused performance).

In Parse’s (2021a) humanbecoming teaching-learning model, “Humans are always on a teaching-learning journey of giving-receiving in mutual process with predecessors, contemporaries and successors in cocreating becoming” (p. 180). This view identified that students come with knowledge and past experiences that they bring to the teaching-learning situation. Through dialogue with students, the different experiences, perspectives, and values related to the current specific context are articulated and may shift with new knowledge, “transforming the meaning with the becoming visible–invisible becoming of the emerging now in contextually construed situations” (Parse, 2021a, p. 180).

A literature review was conducted on the phenomenon of living on the edge. Although living on the edge has been explored from many disciplinary perspectives, there were no identified studies considering living on the edge from a nursing teaching-learning perspective, although it has been written about theoretically. Bunkers’ dialogue and reflections on developing the *NSQ* teaching-learning column (Yancy, 2017) provided insights “in engaging with others in coming-to-know” (p. 25) and “pushing the edge and that is where change occurs is on the edge” (p. 27), creating opportunities and simulating teaching-learning possibilities within the academy. Investigating students’ experiences of living on the edge in nursing education is a study that enhances understanding of the participants’ lived experiences, actions, meanings, perceptions, values, and goals from their perspectives. This study was designed to enhance understanding of the meaning of living on the edge from students who have lived it. With no previous literature focused on living on the edge from a teaching-learning perspective, the humanbecoming teaching-learning model (Parse, 2004, 2021a), specifically the teaching-learning processes (Tables 1 and 2), guided the search for this literature review to synthesize prior research (ambiguity draws attention to uncertainty and complexity; mystery to the fears and vulnerabilities of the unknown), identifying gaps in existing knowledge and providing direction for this research to extend discipline-specific knowledge of humanbecoming teaching-learning processes.

**Table 1.** The Humanbecoming Teaching-Learning Model: The Sciencing Art of Teaching-Learning.

Essences	Paradoxes	Processes
*Semantic cohering* is living with ambiguity in rationaling-intuiting amid clarifying-obscuring in appreciating mystery.	*Rationaling-intuiting* is coming-to-know something that informs human understanding. *Clarifying-obscuring* is struggling with the utterable and unutterable in glimpsing illuminating moments.	*Living with ambiguity* is moment-to-moment moving with the vague. *Appreciating mystery* is apprehending with awe the unfathomable.
*Synergistic patterning* is potentiating integrity in warping-woofing amid the ebbing-flowing of weaving illimitably.	*Warping-woofing* is shaping fabrics of meaning. *Ebbing-flowing* is gliding with the emerging now.	*Potentiating integrity* is strengthening a oneness of purpose. *Weaving illimitably* is unbounded interlacing.
*Aesthetic innovating* is honoring wisdom in considering-composing amid beholding-refining in witnessing unfolding.	*Considering-composing* is pondering possibles. *Beholding-refining* is cautiously cultivating.	*Honoring wisdom* is revering sagacity. *Witnessing unfolding* is profoundly attending with the everchanging.

*Note.* From *The Humanbecoming Paradigm: An Everchanging Horizon*, by R. R. Parse, 2021a, Discovery International, p. 181.

**Table 2.** The Humanbecoming Paradigm.

Assumptions	Postulates	Principles	Concepts and Paradoxes
Humanuniverse is indivisible, unpredictable, everchanging. Humanuniverse is cocreating reality as a seamless symphony of becoming. Humanuniverse is an illimitable mystery with contextually construed pattern preferences. Ethos of humanbecoming is dignity. Ethos of humanbecoming is august presence, a noble bearing of immanent distinctness. Ethos of humanbecoming is abiding truths of presence, existence, trust, and worth. Living quality is the becoming-visible–invisible-becoming of the emerging now. Living quality is the everchanging whatness of becoming. Living quality is the personal expression of uniqueness.	Illimitability is the indivisible unbounded knowing extended to infinity, the all-at-once remembering-prospecting with the emerging now. Paradox is an intricate rhythm expressed as a pattern preference. Freedom is contextually construed liberation. Mystery is the unexplainable, that which cannot be completely known unequivocally.	Structuring meaning is the imaging and valuing of languaging.	*Imaging:* explicit-tacit; reflective-prereflective *Valuing:* confirming–not-confirming *Languaging:* speaking–being-silent; moving–being-still
		Configuring rhythmical patterns is the revealing-concealing and enabling-limiting of connecting-separating.	*Revealing-concealing:* disclosing–not-disclosing *Enabling-limiting:* potentiating-restricting *Connecting-separating:* attending-distancing
		Cotranscending with possibles is the powering and originating of transforming.	*Powering:* pushing-resisting; affirming–not-affirming; being–non-being *Originating:* certainty-uncertainty; conforming–not-conforming *Transforming:* familiar-unfamiliar

*Note.* From *The Humanbecoming Paradigm: An Everchanging Horizon*, by R. R. Parse, 2021a, Discovery International, p. 34.

Limited nursing education research has been completed on teacher-student pedagogies using nursing theory as a foundation due to various factors, including but not limited to lack of instructor incentive, funding, resources, and motivation to move to a new pedagogy (Diekelmann, 2005). Lack of evidence for and the difficulty in validating a nursing theory–based pedagogy continues to put professional nursing education and the public “at risk,” which is a genuine and ongoing challenge (Diekelmann, 2005). The intent of Benner and colleagues’ (2014) work was to assist and inspire educators to use “innovative pedagogical strategies that integrate knowledge acquisition, knowledge use, and engaged attuned reasoning as the situation changes across time” (p. 545). In summary, teachers may respond more individually and effectively by better understanding the students’ perspectives through research and analyzing the meaning of their lived experiences through the lens of the humanbecoming teaching-learning model.

Reflecting on the reviewed literature, several challenges remain in the ongoing knowledge-to-practice gap (Hickerson, et al, 2016; Kavanagh & Szweda, 2017; Wimmers & Mentkowski, 2016). It has been suggested that nursing students are inadequately trained in critical thinking and decision-making, and curricular changes have not occurred at the magnitude needed (Jowsey et al., 2020; Kavanagh & Szweda, 2017). The shift to learner-centered and engaged learning has not occurred to the degree anticipated (Bain, 2004; Benner et al., 2014; Katznelson et al., 2018). The student’s role in achievements is often not acknowledged (Hills & Levitt Jones, 2017; Paterson, 2007), and there continues to be limited nursing education research completed on pedagogies with a lack of nursing theory–based pedagogy (Benner, 2010, 2014; Diekelmann, 2005).

There remain opportunities to enhance understanding of student perspectives through studying their lived experiences in teaching-learning. Using the humanbecoming teaching-learning model as a lens, teachers may be guided to respond more individually and effectively to the complexities of students’ learning experiences to advance nursing knowledge while enhancing understanding of the humanbecoming teaching-learning model and its relevance to the teaching-learning experience. The final opportunity is to provide a solid foundation (based on philosophy, education, and nursing research) to complement and enhance understanding of the humanbecoming paradigm (Parse, 2021a).

## Phenomenon of Interest

The researcher was interested in studying the lived experience of living on the edge for nursing students in the teaching-learning context. Living on the edge is inherent in the day-to-day, in choices and decisions regarding each situation. There is no unique definition for this phenomenon in society; however, common usage suggests that risk, ambiguity, change, and challenge without guaranteeing reward or success are elements of living on the edge.

## Research Question

The research question for the study was, What is the general structural description (or meaning) of the lived experience of living on the edge for nursing students? The interrogatory statement guiding students’ responses was, “Please write about a teaching-learning situation or experience in your nursing program that depicts what living on the edge is like for you.”

## Research Method

To uncover what the students’ lived experiences were like while living on the edge in teaching-learning, Giorgi’s descriptive phenomenological method (Giorgi, 1985, 2009) enabled a greater depth of exploration into their unique experiences. The theoretical perspective that guided the research process and the analysis-synthesis of the findings was Parse’s (2004, 2021a) humanbecoming teaching-learning model, underpinned by Parse’s (2021a) humanbecoming paradigm.

The research question determined the method used to collect the data. The decision to take advantage of online technologies available to nursing students within the academic community was pragmatic, as it ensured up-to-date and accurate email addresses within this environment and drew on participants’ technology-savvy skills (Mason & Ide, 2014). The use of asynchronous communication technology (Limesurvey) allowed participants to explore and revisit their insights when developing their responses, allowing them to move back and forth through their narrative descriptions, thinking about their responses, and drafting and redrafting what they wanted to include and highlight (Mann & Stewart, 2000; Neville et al., 2016). The university research ethics board approved this study, and I ensured that all nursing students from all sites were invited to participate.

### Participant Selection and Ethical Considerations

With this method, the students self-selected to participate, and as a result, the participants may not be representative of the student population (Neville et al., 2016). Email submissions were anonymous, and only I could access the descriptions, which were password-protected. The recruitment process occurred over 12 months during the COVID pandemic, and potential participants were invited with e-posting to participate, which included information on the research contribution, relationship, and benefits of the study. Participants responded anonymously if they chose to participate, implying consent. Confidentiality and anonymity needed to be guaranteed to the participants, as it was essential for them to feel safe enough to respond and freely write about their experiences, values, beliefs, and feelings. Parse (1987) suggested that an acceptable number of participants for this type of research method are 2 to 10 persons who are living the experience. Giorgi (1985, 2009) suggested that the sample size did not need to be large. Participants were nursing students enrolled in a Canadian university nursing program who chose to respond to an invitation: “Please write about a teaching-learning situation or experience in your nursing program that depicts what living on the edge was like for you.”

## Analysis-Synthesis Process

The findings for this research study are presented through the theoretical lens of the humanbecoming paradigm (Parse, 2021a) and, more specifically, the humanbecoming teaching-learning model (Parse, 2004, 2021a). This research illuminates students’ perspectives and contributes new knowledge to the growing body of literature on nursing-specific pedagogies envisioned to enhance understanding of the phenomenon of living on the edge in teaching-learning from the students’ perspectives and offers recommendations for teaching-learning that support interdisciplinary teaching-learning practices in the development of resilient, self-directed, lifelong learners.

The analytic research processes used in Giorgi’s method to arrive at the research findings are identified and described with linkages from the findings to direct participant quotations and excerpts of the participants’ descriptions. The analysis-synthesis consisted of six steps. First, the participant experiences were read and reread for a sense of the whole experience, and the participants’ words were separated into meaning units. The meaning units were transformed to focal meaning, which is at a higher level of abstraction using the researcher’s language. This was followed by a synthesis of the focal meanings into a structural description for each participant. Structural descriptions were then synthesized into one general structural description for all participants, which depicts the central findings of living on the edge in teaching-learning for these participants.

### Reading for Sense of the Whole

Nursing student participants provided three descriptions, but only one was chosen due to the word limit. The first step of the phenomenological process was to dwell with the description as it was presented, reading each in a natural everyday attitude for a sense of the whole (Giorgi, 1985, 2009) teaching-learning experience. The meanings were moments within the lived experience, so they could not stand alone but were considered within the context provided by the participant. In reading and rereading, it was essential to be present with participants’ descriptions to uncover the expressed meanings, neither adding to nor taking away from what was given (Giorgi, 2009). The approach to uncovering the meaning of the lived experience for the nursing student participants of living on the edge in a teaching-learning experience and the descriptive processes used are depicted in Table 3 for one participant’s description.

**Table 3.** Example of Natural Meaning Units and Two Focal Meanings for Taylor.

Meaning Unit	Focal Meaning
To me, living on the edge in nursing means I am in the perfect state for growth. It means I am comfortable enough to have confidence and not be completely overwhelmed, but also that I am very close to the unknown.	Living on the edge meant being in a state of equipoise of sureness-unsureness (living with ambiguity) in speaking–being-silent, and moving–being-still with the fortitude to venture onward (potentiating integrity) with envisioning the not-yet known (appreciating mystery).
By living on the edge as a nursing student, I am able to constantly acquire new information, while also being grounded enough not to fall off and become overstressed or too uncertain to make decisions.	Heightened experience of knowing–not-knowing (living with ambiguity) when configuring new meanings (honoring wisdom) in the light of certainty-uncertainty of confirming–not-confirming when envisioning possibilities (witnessing unfolding) demonstrates evidence of living on the edge.

### Identifying Meaning Units

In identifying meaning units, the participant’s words are changed into third-person rather than first-person expressions. “This makes it clear that the researcher is analyzing another’s experience rather than one’s own” (Giorgi, 2009, p. 153). With this transition to the third person, I was able to dwell with the lived experience of another, minimizing the making of comparisons to previous knowledge or my own experiences. The identification of meaning units made my analysis of the description of the lived experience more manageable. It required the identification of transitions in meaning, which identified significant ideas. In the process of dwelling with the lived experience of others, I was “analyzing the experiences of others, so in order to avoid the fusion between the researcher and the participant’s experiences, all first-person statements are changed into third person statements [using nonbinary pseudonyms]—but otherwise remain the same” (Giorgi et al., 2017, p. 180).

### Identifying Focal Meanings and Transforming Meaning Units

Uncovering focal meanings required dwelling with the meaning units transformed to a higher level of discourse (Table 4). In seeking focal meanings, free imaginative variation (Giorgi, 2009) and reflection were critical to determining those essential characteristics of the lived experience of living on the edge in teaching-learning within the participant descriptions. The words of the participants were shifted into expressions that are more directly relevant to the humanbecoming paradigm and humanbecoming teaching-learning model. In dwelling with the participants’ language, the humanbecoming concepts and paradoxes (Parse, 2021a, p. 34) surfaced along with the humanbecoming teaching-learning processes. Elevation to this new level of abstraction was guided by the humanbecoming ontology and humanbecoming teaching-learning model, which provided the opportunity to generalize the experience so it was no longer situation-specific and could be integrated with other descriptions. The synthesis of the situated structure of the experience is a descriptive paragraph that specifies the lived experience of living on the edge through the lens of the humanbecoming teaching-learning model for each participant. “This structure is meant to depict the lived experience of a phenomenon, which may include aspects of the description of which the experiencer was unaware” (Giorgi, 2009, p. 166).

**Table 4.** Focal Meanings for Taylor.

A. Living on the edge meant being in a state of equipoise of sureness-unsureness, living with ambiguity while demonstrating the fortitude to venture onward (potentiating integrity) with envisioning the not-yet known (appreciating mystery).
B. Heightened experience living with ambiguity when configuring new meanings and (honoring wisdom) when envisioning possibilities (witnessing unfolding) demonstrates evidence of living on the edge.
C. Living on the edge was knowing that emerged in the first performance of a skill. Taylor experienced cautiously practicing on a peer (witnessing unfolding), drawing on previous experience (weaving illimitably) when engaging in this new experience, which was affirming.
D. Living on the edge arose with the novel experience that started with feelings of living with ambiguity while doing the familiar in an unfamiliar environment.
E. Living on the edge was being prepared with knowledge yet experiencing the uncertainty of carrying out the skill while cautiously cultivating the opportunity to give an injection, while witnessing the unfolding.
F. In witnessing unfolding, Taylor risked going into the abyss of knowing–not-knowing; there was certainty-uncertainty and the all-at-once revealing-concealing, enabling-limiting, and connecting-separating of living with ambiguity.
G. In appreciating the mystery of the illimitability of nursing knowledge and everchanging demands for ongoing education to ensure safe, competent professional practice, for Taylor, living on the edge means venturing forth to pursue daunting endeavors, while vigilantly attending to the possibilities in becoming.

### Essential Structural Description for Taylor

For Taylor, living on the edge meant being in a state of sureness-unsureness in speaking–being-silent, and moving–being-still with the fortitude to persevere with envisioning the not-yet known, in dwelling with the participant’s words through the lens of the humanbecoming paradigm (Table 2) and the humanbecoming teaching-learning model (Table 1). The participants experienced the humanbecoming concepts and paradoxes (Table 2) of explicit-tacit knowing when configuring new meanings considering certainty-uncertainty in conforming–not-conforming when persevering in new learning experiences. This participant drew on previous experiences, which were affirming, yet experienced pushing-resisting in doing the familiar in an unfamiliar environment. In attending to the everchanging, this participant risked going into the abyss of knowing–not-knowing; there was certainty-uncertainty and the all-at-once enabling-limiting of living quality as a nurse. This participant was committed to the vision of the illimitability of nursing knowledge and everchanging demands for ongoing education to ensure safe, competent professional practice. Living on the edge meant venturing forth and pursuing daunting endeavors while vigilantly attending to the possibilities of becoming but did not mean being unsafe or irresponsible.

### Synthesizing a General Structural Description

The general structural description of living on the edge in teaching-learning emerged from synthesizing the three situated structural descriptions provided (Table 5). Although the situated structural descriptions remained faithful to the participants’ descriptions of their lived experiences and were situation-specific, “the general description of the situated structure tries as much as possible to depart from the specific to communicate the most general meaning of the phenomenon” (Giorgi, 1985, p. 20). This structure is meant to depict the lived experience of the phenomenon of living on the edge in teaching-learning: “It means that in principle, the structures are applicable to more individuals than the persons upon which they were based” (Giorgi, 2009, p. 166). “Writing of the structure takes a much more holistic perspective than the transformations themselves required” (Giorgi, 2009, p. 167). To gain insight into the essences of the lived experiences, I intentionally dwelt with each of the descriptions, moving to and fro, to synthesize and distinguish the invariant and key meanings of the lived experience of living on the edge in teaching-learning. The humanbecoming teaching-learning model processes (Table 1) were used to guide the synthesis of the lived experience and to craft a careful description containing the constituent meanings and the relationships among the essential meaning units of the experience. The general structural description conveys what was essential within this series of teaching-learning experiences, transcending the situations from which they were obtained (Giorgi, 2009).

**Table 5.** Comparison Table of Situated Structural Descriptions.

Humanbecoming Teaching-Learning Model Essences	
Semantic cohering is living with ambiguity in rationaling-intuiting amid clarifying-obscuring in appreciating mystery (Parse, 2021a, p. 181).	
Reilly	In living on the edge in teaching-learning, Reilly experienced disquiet with the paradoxical rhythms of clarifying-obscuring while shaping personal knowing in persevering the cherished endeavors. In rationaling-intuiting, Reilly structured meaning imaging new possibilities.
Taylor	For Taylor, living on the edge meant clarifying-obscuring with the fortitude to persevere with envisioning the not-yet known. In rationaling-intuiting, Taylor experienced glimpses of knowing when configuring new meanings and envisioning possibilities.
Quinn	Quinn experienced that rationaling-intuiting in being with and bearing witness to a client experiencing possible medical complications through in-person simulation was clarifying-obscuring in coming-to-know something of value.
Synergistic patterning is potentiating integrity in warping-woofing amid the ebbing-flowing of weaving illimitably (Parse, 2021a, p. 181).	
Reilly	Reilly experienced surprise and dismay when an honored other disregarded Reilly with disrespectful comments directed to Reilly as a person, shattering Reilly’s hopes and dreams. Reilly experienced shame that arose through dishonoring experiences with others in the struggle for personal and professional identity. Reilly struggled when choosing to live value priorities, potentiating integrity in affirming intentions in light of non-being. Reilly was weaving illimitably with others (professors and peers), being with yet apart all at once; this ebbing-flowing surfaced with feelings of anguish that arose with the overwhelming adversity of academic and private challenges.
Taylor	Taylor experienced warping-woofing when persevering in a new learning experience, drawing on previous experience that was affirming. Taylor experienced ebbing-flowing in doing the familiar in an unfamiliar environment, cautiously cultivating savoring the new experience.
Quinn	Quinn shared that the rhythms of ebbing-flowing with the simulated learning experience were so authentic that Quinn was immersed in moving with meaning of the experience. Quinn observed that once the simulation activities were completed, they focused on warping-woofing and shaping meaning in their experience(s), transforming their knowledge of theory to practice.
Aesthetic innovating is honoring wisdom in considering-composing amid beholding-refining in witnessing unfolding (Parse, 2021a, p. 181).	
Reilly	With a revered other’s authentic presence, Reilly found voice in considering-composing a new pathway in what was valued in choosing pattern preferences. In beholding-refining, Reilly overcame adversity and experienced a sense of well-being. In beholding-refining, while transforming and imagining a new view of self, relationships, and projects, Reilly was confirming cherished value priorities. For Reilly, the lingering presence of “living on the edge” was persevering with cherished endeavors amid the paradoxical tensions that existed in nursing academia.
Taylor	In considering-composing, Taylor risked going into the abyss of knowing–not-knowing; there was certainty-uncertainty and the all-at-once enabling-limiting of living quality as a nurse. Taylor committed to the vision of beholding-refining the illimitability of nursing knowledge and everchanging demands for ongoing education to ensure safe, competent professional practice. For Taylor, living on the edge meant venturing forth pursuing daunting endeavors, while vigilantly attending to the possibilities in becoming. It did not mean being unsafe or irresponsible.
Quinn	Quinn considered the simulation to be an affirming learning approach that enabled their ability to move beyond in their knowledge, skills, and abilities and cotranscending ways of beholding-refining. A lingering presence arose in considering-composing of the enduring of truths that were uncovered in the simulation, transforming what was learned in a new light.

### The General Structural Description

After I carefully dwelt with the various facets of the individual situated structural descriptions and viewed them through the lens of the humanbecoming teaching-learning model (Parse, 2004, 2021a), the central findings or general structural description of living on the edge in teaching-learning for these nursing student participants emerged.

Living on the edge in teaching-learning is

- acquiescing to ambiguity in the paradoxical rhythms of moving moment-to-moment, coming-to-know something of value;
- dignifying the journey by vigilantly exploring unknowns of the everchanging;
- risking venturing forth with quieting-disquieting regard amid diverse alliances (ideas, objects, experiences, and others) in anticipation of cherished endeavors;
- persevering with novel experiences amid potentiating-restricting opportunities in realizing cherished endeavors;
- venerating words, wisdom, and images of others in considering-composing cherished choosings; and
- discovering anew cherished priorities as possibilities emerge.

## Discussion of the Findings

The research findings yielded three unique, substantial, yet discrete teaching-learning lived experiences. Within participant Reilly’s description of living on the edge in teaching-learning, there was discordant communication with Reilly regarding a career path. As the experience unfolded, clarity and awareness of choices became more precise in hindsight. Within this teaching-learning description are the concepts of reflecting on a new view (imaging), confirming cherished value priorities (valuing), and recognizing future career goals (languaging); the paradoxes of revealing-concealing, enabling-limiting, and connecting-separating were apparent rhythms within this lived experience for Reilly. Reilly pushed forward (powering) in the face of certainty-uncertainty (originating), eventually finding new ways of helping others (transforming).

In Taylor’s written description, living on the edge in the teaching-learning experience focused on structuring meaning in imaging, valuing, and languaging of acquiring knowledge, skills, and abilities on this lifelong learning journey. The goals were identified within the teaching-learning process. Taylor expressed unsureness and discomfort at various points in coming-to-know, demonstrating the rhythmical patterns of revealing-concealing, enabling-limiting, and connecting-separating in creating a preferred preference and balance to achieve learning goals. In cotranscending with possibles, Taylor recognized that real-life situations are messier and more intricate than our conscious presence. Taylor identified the need to make an intentional effort (powering) to go into those complex and unknown spaces (originating) in order to continue learning and advancing practice (transforming).

Quinn’s description focused on being with and bearing witness to a client experiencing possible complications during a meaningful in-person simulation that was immersive (imaging, valuing, and languaging) and engaging (powering) in structuring meaning to support learning. Quinn shared that the experience was so natural and authentic that they felt immersed (living rhythmical patterns revealing-concealing, enabling-limiting, and connecting-separating) in a real clinical environment (originating) in coming-to-know (transforming) something of value (valuing).

According to Giorgi and colleagues (2017), “There are no ‘perfect descriptions’: there are good ones, adequate ones and inadequate ones. The good ones allow for a rich analysis of the experience, and the adequate ones will allow a structure to be developed” (p. 183). The general structural description of living on the edge in teaching-learning for the nursing student participants of this study contained six essential themes or essences.

### Reflections on Essences

#### Acquiescing to ambiguity in the paradoxical rhythms of moving moment-to-moment, coming-to-know something of value

Accepting ambiguity is necessary today, as “yet-to-be-discovered answers to serious questions continue to unfold, as explicitly living with ambiguity becomes more and more commonplace” (Parse, 2021c, p. 5). Each participant described aspects of ambiguity in the paradoxical rhythms of comfort-discomfort, certainty-uncertainty, and confirming–not-confirming. For Taylor, living on the edge in teaching-learning meant being well situated with a balance of certainty-uncertainty and imaging explicit-tacit and reflective-prereflective moments with the fortitude to explore the unknown. Taylor found the research question ambiguous, which created uncertainty, but chose to venture forward in exploring the unknown. For Taylor, living on the edge meant being in a state of equipoise of sureness-unsureness in speaking–being-silent and moving–being-still with the fortitude to venture onward with envisioning the not-yet-known.

*I found the question vague but decided I would answer it anyway; maybe that was intended. To me, living on the edge in nursing means I am in the perfect state for growth. It means I am comfortable enough to have confidence and not be completely overwhelmed but also that I am very close to the unknown.—Taylor*

Yancey (2018) noted that “creating a teaching-learning space where students are comfortable in acknowledging not knowing is essential in illuminating a path where students can learn to let go of thinking they need to be the all-knowing-expert” (p. 229). All participants acquiesced to the ambiguity of the experience, moving with the paradoxical rhythms of moving moment-to-moment in coming-to-know something of value.

#### Dignifying the journey by vigilantly exploring unknowns of the everchanging

Dignifying the journey recognizes the mystery that permeates our lives; we choose internal, relational, and pragmatic “ways of living with conforming–not-conforming and certainty-uncertainty as what lies on the path of creating a new way is not known” (Bunkers, 2019, p. 175). The participants each dignified the journey by appreciating mystery in shaping their personal knowing and structuring meaning. Each participant described their journey of discovery, exploring their unknowns and envisioning their path forward. Reilly experienced surprise and dismay when an honored or privileged other disregarded Reilly with comments that shed a negative light on Reilly as a person, shattering Reilly’s hopes and dreams. Reilly described a difficult conversation with a professor:

*I remember there was a moment in my first year of the nursing program when one of my professors said something that was very unkind towards me and absolutely astonished me that somebody that was a nurse and a teacher could say that to somebody. She told me outright to my face that “it would be best off if you left the program willingly. You don’t have what it takes to become a nurse.”—Reilly*

Parse (2021a) defined semantic cohering as “living with ambiguity in rationaling-intuiting amid clarifying-obscuring in appreciating mystery” (p. 181). Each participant described the experience in accepting ambiguity in coming-to-know something that informed understanding and dignified the teaching-learning journey by vigilantly exploring unknowns in the “utterable and unutterable in glimpsing illuminating moments” (Parse, 2004, p. 35).

#### Risking venturing forth with quieting-disquieting regard amid diverse alliances (ideas, objects, experiences, and others) in anticipation of cherished endeavors

Reilly expressed surprise and dismay when an honored other’s comments disrespected Reilly as a person and shattered Reilly’s hopes and dreams. Reilly chose to risk living chosen value priorities, pushing-resisting of affirming–not-affirming intentions considering being–non-being. In Bunkers’ (2009) phenomenological-hermeneutic exploration of the living experience, “Taking a risk is venturing forth amid potential peril, as apprehension with elation surfaces in novel engagements” (p. 240). For Taylor, living on the edge meant performing a new skill on a patient for the first time. Foundational knowledge and practical experience in the skills laboratory prepared Taylor to perform this skill. Living on the edge arose with Taylor’s novel experience of pushing-resisting in doing something new yet familiar in an unfamiliar environment, which resulted in feelings of certainty-uncertainty. Taylor was prepared with knowledge yet experienced the familiar-unfamiliar of carrying out the skill.

*The living on the edge part came when I needed to do something new, which was giving an injection to a patient in the hospital. I had never done that before and thus was nervous. Because I had a solid grounding state and also some new unexplored territory, I was living on the edge and I was in the perfect position to learn and grow my skills. To me, living on the edge is a necessary balance.—Taylor*

The participants spoke of risking venturing forth with quieting-disquieting regard amid diverse alliances in anticipation of cherished endeavors. Participants described their unique experiences of the paradox of quieting-disquieting regard amid their diverse alliances, which demonstrated living pattern preferences of pushing-resisting, affirming–not-affirming, and confirming–not-confirming as new possibilities and meanings emerged.

#### Persevering with novel experiences amid potentiating-restricting opportunities in realizing cherished endeavors

“Novel engagements arise from imagination and ingenuity. Novel engagements are projects, ideas, and people that challenge and offer adventure. Novel engagements involve the interconnection of unique individuals and events” (Bunkers, 2009, p. 246). “Persevering through a difficult time can be seen as persistently pursuing a course of action despite obstacles” (Allchin-Petardi, 1998, p. 172). In a study to gain an understanding of the lived experience of persevering through a difficult time, Allchin-Petardi (1998) found that “the lived experience of persevering through a difficult time is deliberately persisting with significant engagements while shifting life patterns” (p. 174). In Taylor’s words,

*If I go too far into unknown territory (which is “falling off the cliff”), then my brain is in no state to learn and remember anything. If I don’t get close enough to the edge, then I will not be able to improve as a nurse.—Taylor*

Each participant spoke of engaging in novel experiences and the rhythms of potentiating-restricting that unfolded in persevering with prospects. Participants were living quality in the becoming visible-invisible becoming of the emerging now and the core knowings of fortifying wisdom, discerning witness, and penetrating silence (Parse, 2021b). They were fortifying wisdom through the rhythms of clarifying-obscuring, sureness-unsureness, and propelling-opposing. While discerning witness arose in the emergence of new knowledge through the paradoxes of seeing – unforeseeing, differentiating-non-differentiating and exposing-hiding (Parse, 2021b). Penetrating silence occurred in moments of enlightenment and discovery in their teaching-learning journey, reflecting easing-uneasing, illuminating-eclipsing and inventing-impeding (Parse, 2021b).

#### Venerating words, wisdom, and images of others in considering-composing cherished choosings

Living on the edge is venerating words, wisdom, and images of others in considering-composing the cherished. Revering the unfolding moments of the teaching-learning journey is to be present, and “inviting dialogue is a way of honoring the wisdom of others” (Parse, 2014, p. 5). Reilly described a valued other’s authentic presence, which enabled Reilly to disclose–not-disclose explicit-tacit knowings and all-at-once, uncovering a new pathway in confirming–not-confirming what was valued in choosing pattern preferences. Parse (2004) identified that “potentiating integrity is strengthening a oneness of purpose. The teaching-learning process is fortified where intentions are respected” (p. 35). Taylor committed to the vision of the illimitability of nursing knowledge and everchanging demands for ongoing education to ensure safe, competent, professional practice. “Fortifying wisdom is strengthened with the intuitive insights arising with sureness-unsureness of intensely propelling with the unexplainable winds of ambiguity of living quality” (Parse, 2021b, p. 58). Taylor recognized that there are unlimited learning opportunities that are necessary for maintaining professional competence.

*In nursing, you need to constantly improve just to stay in the same place. The science and best practices are constantly changing; there are always new opportunities to improve your ability to care. If you are not moving forward (and you move forward/improve by living on the edge), then you are essentially moving backward. As a nurse, if I am not constantly trying to improve my care, then I am reducing my competencies as a nurse. Other examples of living on the edge: keeping up with the latest research of evidenced-based practice and applying it to my care.—Taylor*

All three participants described venerating words, wisdom, and images of others in considering-composing the cherished. Each participant was attentive and responsive to the everchanging, listening intently as the journeys unfolded. The paradoxes of explicit-tacit knowings, connecting-separating in confirming–not-confirming occurred with honoring-dishonoring in attending distancing on their unique learning journeys.

#### Discovering anew cherished priorities as possibilities emerge

“Discovering is noticing what is in the depth of stillness in the immersion of penetrating silence. The noticing is personal discerning, the foreshadowed with the inherent affirming–not-affirming of being–non-being in profoundly searching for different ways of becoming” (Parse, 2021b, p. 58). For Taylor, living on the edge was venturing forth to pursue daunting endeavors while discovering new cherished priorities as new learning opportunities emerged. For Taylor, living on the edge did not mean being unsafe or irresponsible. In choosing to be available and open to challenging and uncertain situations, Taylor discovered anew, mastering and learning without crucial ramifications for themselves or others. Taylor believed that nurses are ethically responsible for engaging in difficult conversations with patients, which involves living on the edge. It does not mean being risky, unsafe, careless, or negligent but instead reflects the paradox of attending-distancing in being present and supportive.

*It doesn’t mean being dangerous or reckless. Rather, it means the constant intentional effort to place myself in a difficult and unknown situation that I believe I will be able to conquer and learn from without serious consequences for myself or others —Taylor*

The relevant literature consistent with the humanbecoming paradigm and teaching-learning model was selected and woven through this discussion of essences with linkages to the participants’ words. Upon reflection, living on the edge is a dynamic experience that requires an openness to change and courage to navigate between perceived opposites (paradoxes).

### Contributions of this Study

This small group of participants, a convenience sample, represented the nursing student population; generalizability was not the goal, as we can never fully understand what another is sharing because we interpret it in our own way, based on our own experiences, values, and beliefs. This study provided an opportunity to explore living on the edge in teaching-learning and inform understanding of the experience. “Witnessing is non-intrusive, gentle glimpsing in reaching beyond to honor the other is human dignity” (Parse, 2021a, p. 118). Further research would be valuable to further explore nursing students’ unique and diverse experiences.

Although numerous studies have been conducted addressing the teaching of nursing students (methods and models), notably absent from this research are the more individualized, holistic perspectives. I have attempted to capture the essence of the lived experience of living on the edge in teaching-learning and to describe the students’ perspectives and meanings of those lived experiences. The analysis revealed a general structural description, including six essences applicable to all three participants’ lived experiences in teaching-learning. This study successfully captured the perceptions of these nursing students as individual, unique human experiences in teaching-learning, encompassing personal goals, hopes, dreams, and wishes and how the participants were transformed. These authentic descriptions provided a foundation to foster an understanding of the nursing student experience and its uniqueness to each nursing student. The study’s conclusions are related to ongoing theory development, further research, and practice.

### Observations

In nursing, we tend to borrow from and explore questions using other disciplinary knowledge, as I have done in this interdisciplinary doctoral research approach (adult education, philosophy, and nursing). Giorgi’s descriptive phenomenological research method (2009) was chosen as the method, and experiences were viewed through the lens of Parse’s humanbecoming teaching-learning model (2021a) and humanbecoming ontology (2021a), supported by my interdisciplinary doctoral committee. This research highlights the significance of nursing theory and the relationships between theory, research, and practice. It highlights the importance of the discipline of nursing maintaining its distinct knowledge, using a dynamic, living metatheory or paradigm. Using nursing theory and research to guide teaching-learning practice can increase capacity, capability, resilience, and grit (Duckworth, 2016) so students can transcend and reach their fullest potential. Describing the meaning and essence of the lived experience of living on the edge in teaching-learning guides academia, future research, and practice by making explicit the learning processes and recognizing that learning and structuring meaning are both personal and relational.

As I reflect on the written descriptions of these experiences and the observations that participants shared of their discoveries and understandings of their situations, I see that their interpretations may not be what they envisioned. The participant descriptions were written from their frames of reference, experiences, values, and beliefs, which are all central to their humanbecoming. The participants freely interpreted and integrated information into their own frames of reference, and those interpretations became part of their ways of being. Individuals (students, teachers, or peers) can never fully understand one another, so to some degree, there is always a living with the tension of being understood-misunderstood in relationships with others. Hence, teachers need to consider their own words and actions carefully because words and ways of being are so important to how others might interpret what was said and done. The humanbecoming teaching-learning model with essences and processes was a guide in developing teaching-learning strategies that contribute to excellence in nursing practice. There was an appreciation for living with ambiguity and the significance of appreciating mystery of nursing practice within the teaching-learning process, being attentive, and bearing witness and weaving illimitably with participants’ descriptions of how they moved toward their cherished endeavors potentiating integrity in this study. In witnessing unfolding, there was an honoring of wisdom and attentiveness to the everchanging perspectives while pondering teaching-learning possibilities. The uniqueness of each participant’s written descriptions, similarities, and differences has been presented, and paradoxical rhythms of the humanbecoming teaching-learning processes are explicated as viewed through the lens of the humanbecoming ontology.

### Implications for Nursing and Nursing Education

#### Acquiescing to ambiguity in the paradoxical rhythms of moving moment-to-moment, coming-to-know something of value

We (teachers) must encourage learners to accept, embrace, and appreciate the opportunities that ambiguity provides for meaningful exploration, which may create discomfort. There is a need to reinforce that new graduates are members of a community and that all cannot be known or fully understood. Key in each of these transitions is the need to accept varying degrees of ambiguity and to understand that they are not expected to be experts but rather engaged learners.

#### Dignifying the journey by vigilantly exploring unknowns of the everchanging

In acquiring new knowledge, exploring novel experiences, and developing new skills, it is essential to recognize that individuals are mysteries and can never be fully understood. Each client will respond uniquely to care, disease processes, and treatments. Nursing students need to develop a comfort level for the unexpected and unanticipated responses of real life, moving away from linear thinking of cause and effect to “learning to live with the gray.”

#### Risking venturing forth with quieting-disquieting regard amid diverse alliances (ideas, objects, experiences, and others) in anticipation of cherished endeavors

There is always a risk of being disregarded in professional practice by others (Ursel & Aquino-Russell, 2010). Learning in a safe environment allows one to address challenges intentionally. In-person or immersive virtual simulations provide the opportunity to create a safe environment to address these challenges intentionally. Nursing students have identified challenges in person-centered care, difficult conversations, and moving with the unanticipated and unpredictable.

#### Persevering with novel experiences amid potentiating-restricting opportunities in realizing cherished endeavors

Working with students to develop and enhance resilience is key in their ability to address bullying issues and being disrespected and in learning to maintain a “balance” in their knowing–not-knowing and capability to advocate for themselves to address their unique learning needs and knowledge gaps. Building resilience may help students to persevere in academia and their future professional practice. The experience of not-knowing, of lacking knowledge to cope with situations provoked by a crisis, is a potential point of entry for learners to reflect on current ways of knowing and being in the world and to engage in changing these ways. A graduate nurse’s transition into a professional role can be an overwhelming experience due to the many new demands. The importance of support as students learn and develop resilience is key; this may mean having the opportunity to practice skills repetitively until students develop mastery and confidence and understand the subtleties of real-world impact on this development.

#### Venerating words, wisdom, and images of others in considering-composing cherished choosings

Teachers and learners need to honor the wisdom and value priorities of all present (students, peers, patients, families, the healthcare team) in this teaching-learning journey (Ursel & Aquino-Russell, 2010). There is a need to instill the importance of lifelong learning to maintain current knowledge and ensure safe, confident, competent, and resilient practice.

#### Discovering anew cherished priorities as possibilities emerge

Self-directed learning is a core professional concept in nursing, and as new opportunities emerge, individuals are required to intentionally engage in deliberate practices and mastery of various skills to ensure safe, compassionate, and competent practice.

These findings underscore the importance of understanding the complexities of nursing students’ lived experiences. Whether students are engaged in competency-based or outcomes-based education, education approaches must be tailored to the individual student’s learning needs, with more humanistic approaches informed by theory, intentional learning activities, and deliberate practices.

### The Path Forward: Recommendations

Limited nursing education research has been completed on teacher-learner pedagogies using nursing theory as a foundation or lens. Further research using nursing theories will expand nursing theoretical perspectives, increase disciplinary-specific knowledge awareness, and enhance professional influence. Innovative pedagogical strategies that integrate discipline-specific knowledge acquisition, use of critical thinking, clinical reasoning, and clinical judgment are key to being responsive in these increasingly complex environments. Living on the edge in teaching-learning occurs for students at various stages of their learning journey: entering the nursing program, in their capstone courses, and as they transition into their careers. Living on the edge could also be investigated more broadly with different populations as it is a universal living experience. Future research with Parsesciencing inquiry (Parse, 2021a) will expand understanding of humanuniverse living experiences and promote research findings from a nursing perspective to further advance nursing science and disciplinary knowledge.

From the conceptualization through to the conclusion of this study, I have set out to capture the essence of the lived experiences of living on the edge for nursing students. The humanbecoming teaching-learning model underpinned by the humanbecoming paradigm guided this research to uncover the structural meaning of living on the edge in teaching-learning. I hope that these research findings and descriptions of participants’ lived experiences will inspire the desire to gain a more holistic and in-depth understanding of the teaching-learning process, the uniqueness of the lived experience of living on the edge in teaching-learning, and the value of discipline-specific theory and research methods. Sharing these findings at meetings and conferences and in publications will enhance understanding of the complexities of the lived experience of living on the edge in teaching-learning for nursing students.
